# Topology identification in distribution system *via* machine learning algorithms

**DOI:** 10.1371/journal.pone.0252436

**Published:** 2021-06-01

**Authors:** Peyman Razmi, Mahdi Ghaemi Asl, Giorgio Canarella, Afsaneh Sadat Emami

**Affiliations:** 1 Faculty of Engineering, Ferdowsi University of Mashhad, Khorasan Razavi, Mashhad, Iran; 2 Faculty of Economics, Kharazmi University, Tehran, Tehran, Iran; 3 Department of Economics and CBER, University of Nevada, Las Vegas, Nevada, United States of America; 4 Faculty of Electrical and Computer Engineering, Islamic Azad University of Tabriz, East Azerbaijan, Tabriz, Iran; University of Glasgow, UNITED KINGDOM

## Abstract

This paper contributes to the literature on topology identification (TI) in distribution networks and, in particular, on change detection in switching devices’ status. The lack of measurements in distribution networks compared to transmission networks is a notable challenge. In this paper, we propose an approach to topology identification (TI) of distribution systems based on supervised machine learning (SML) algorithms. This methodology is capable of analyzing the feeder’s voltage profile without requiring the utilization of sensors or any other extraneous measurement device. We show that machine learning algorithms can track the voltage profile’s behavior in each feeder, detect the status of switching devices, identify the distribution system’s typologies, reveal the kind of loads connected or disconnected in the system, and estimate their values. Results are demonstrated under the implementation of the ANSI case study.

## 1 Introduction

Traditional planning and design of distribution networks and power distribution grids make use of the so-called "fit-and-forget" strategy [1], whereby networks are minimally monitored, and activation and sensing are insignificantly applied. With the increase in the demand for electricity and the number of end-use users, however, the operation and control of power grids have become more and more complex and challenging. Primary among these challenges are the scantness of the communication infrastructure to provide measurements or dispatch real-time commands; the large-scale permeability of the micro-generators from the fluctuating energy resources such as penetration of wind turbine; the local over-voltage and power-line congestions problems [2], in distributed power generation, in particular within the strongly radial, resistive, low-voltage networks; and the connection of dispatchable loads to the power distribution network, concerning, for example, the problem of plugging in the smart buildings as well as electric vehicles. Modern power distribution grids would experience serious congestion problems in case of non-enforcement of appropriate scheduling and coordination protocols [3,4]. In response to these challenges and issues in the power distribution networks and the need for deeper integration, information, and control technologies, the emphasis has shifted away from the conventional approach and moved toward applying novel ideas and paradigms.

Recently, a large amount of research has been devoted to developing and engineering solutions to such challenges without interrupting the grid reliability and efficiency. Most of these studies consider the question of topology identification (TI) of the power distribution grid a necessary preliminary issue to be dealt with [5,6]. Several methods have been suggested for topology identification (TI) of the power system, including the rule method [7–9], the artificial neural network method [10], the minimum information loss method [11], the normalized Lagrange multiplier method [12], the transfer power flow approach [13,14], and the residual error method [15], among others. However, these techniques may not be immediately utilized for topology identification (TI) of the distribution system due to the absence of real-time measurement in the distribution system. Other techniques, such as the voltage estimation method suggested by [16], fail in the distribution grid with limited sensor data or partially frequent topological modifications [17–20]. A MIQP-based topology identification (TI) method which shows good performance under limited real-time measurement as well as pseudo-measurements data has been put forward by [21,22], based on [23,24], where topology identification (TI) is viewed as a basis for the analysis and calculation of the distribution network such as the state estimation, fault location, network reconfiguration, and power flow calculation [21,22]. A data-driven strategy has been applied to topology identification for distribution networks with low-voltages (LVs), including the load phase connectivity from the energy measurement time series. This strategy applies Principal Component Analysis (PCA) and derives its graph-theoretic interpretation for inferring a steady-state network topology from the smart-meter energy measurements [25]. A topology identification technique based on the multi-agent for outage area in the distribution network has been proposed [26]. According to this method, the distribution of topology information is performed in all intelligent terminals, and therefore, terminals may exchange information by the peer-to-peer network. In case of faults, the master-intelligent terminal in the outage area achieves the topology-units information, which has been stored in other terminals and produces a topology structure of the outage area through the connection of each of them. A procedure of topology identification (TI) has been recognized by means of three types of agents (external topology-calling agents, topology-generation agents, internal topology publishing-agents), resulting in an outage area topology that may be effectively and rapidly produced by their interaction [26]. The topology error identification of the distribution network has also been the subject of investigations [27,28]. However, topology error identification based on the state estimation outputs requires the state estimation that enhances the computation and time period. Moreover, in a power distribution network, the network topology information would be required to have effective operations. Hence, the network connectivity information is not frequently provided at low-voltage levels because of the un-informed modifications, which occur from time to time. It is also well-known that some techniques are suitable for distribution grids based upon selective assumptions. For instance, [29–31] assume the availability of each switch location and seek a proper combination. On the other hand, state estimation-based procedures [32,33] and power flow-based techniques [34] assume admittance matrix availability as well as infrequent topology changes. However, these hypotheses are not fit in the newly re-configured and added distribution network, as knowledge of the circuit breakers or admittance matrix information cannot be available.

The topology identification (TI) problem has been regarded as one of the unknown parameter identification problems, which shows the need for studying the parameter identifiability problem for the network systems. From this perspective, [35] has devised a nonlinear adaptive observer with global convergence for jointly estimating the states as well as the uncertain constant parameters, while [36], following [35], has presented an adaptive observer for a class of nonlinear systems with a general nonlinear parameterization under the assumption of boundedness of the state and unknown parameter of the system.

This paper complements the extant literature by approaching the grid topology’s identification issue *via* machine learning (ML) algorithms. The rest of the paper is organized as follows. In Section 2, a brief review of ML algorithms is discussed. In Section 3, we present and discuss the main results. Finally, Section 5 summarizes our conclusions.

## 2 Materials and methods

### 2.1 Machine learning

Machine learning (ML) can be described as the process through which a machine (i.e., a computer) can learn to perform a task with new data or configurations without the need for re-programming [37–39]. The technique is tailored to uncover structure in complex data and efficiently describe it with minimal parameters [37]. The concept of ML originates from the recognition of the pattern and computational learning theory in the artificial intelligence field [40]. Algorithms developed with ML methods are expected to provide learning to perform a task concerning future data that is not present to the algorithm during the training process [41].

### 2.2 Algorithms grouped by learning style

There are different kinds of algorithms or techniques that can model a mathematical or statistical problem depending on the interaction with experiences or environments, or in general, what is called the input data. In other words, depending on the task and purpose of the problem, we may categorize ML algorithms into different classes. A general typology of ML algorithms based upon the learning styles include, among others, the following [42]: a) supervised machine learning (SML) algorithms; b) unsupervised machine learning (UML) algorithms; c) semi-supervised machine learning (S-SML) algorithms; d) reinforcement machine learning (RML) algorithms. In SML, the algorithmic program is "trained" on a pre-defined set of “training examples,” which then facilitate its ability to reach an accurate conclusion when given new data. In contrast, in UML, the program is given data and must find patterns and relationships therein. The S-SML approach takes advantages of both supervised and unsupervised approaches by the ability to work on labeled and unlabeled datasets. Finally, in an RML setting, which is modeled on reinforcement psychology, an agent repeatedly interacts with the environment: at each time step, the agent (a) observes a state of the environment; (b) chooses an action based on its policy—a mapping from the observed states to the actions to be taken; and (c) receives a reward and observes next state. This process continues until a terminal state is reached. As our main interest in this paper lies in the SML algorithms’ context, we first briefly introduce the basic concepts of this class of ML algorithms.

### 2.3 Supervised learning

The supervised case assumes prior knowledge of the underlying phases of the system and, as such, supervised approaches rule out the possibility of learning unknown phases. Contrary to the supervised case, unsupervised methods learn the structure from the data itself without the need for prior labeling [37–38]. In the supervised learning approach, certain inputs and a group of labeled outputs (targets) are provided as well as a certain output in the training process. The algorithm then uses machine inference to develop a function capable of emulating the process and mapping the new input data to the predicted output. In supervised learning, the input data are called the training data with a given label or real outputs like spam or not-spam or a stock price at a time. In the training process, a model is prepared wherein anticipations are necessary, and it is corrected in case of the wrong prediction. Therefore, the training procedure proceeds until the model obtains the proper precision level on the training data. Classification and regression problems are two important examples of supervised learning [42].

The classification problem aims to categorize existing samples or data into a different kind of class. In this group, the existing target value or output samples(label) are discrete or categorical data, and the modeled machine is called a classifier. At first, in such a problem, by using inputs and their label outputs(targets), a trained machine model is provided, and then the machine predicts new data into a different class. In a regression problem, the target value or existing label is a continuous variable, and the modeled machine is called a predictor, thus for the input variable, the machine predicts their actual target value [42]. We provide the algorithm with a certain input, a certain output, and a group of labeled training data in supervised learning. The algorithm then uses machine inference to develop a function capable of emulating the process and mapping the new data. In other words, supervised learning algorithms aim to model the association of target prediction output with input characteristics to enable us to predict output values for novel data according to the mentioned associations those relationships that it has learned from earlier datasets. Examples of supervised machine learning algorithms include: 1) K-Nearest Neighbors (KNN); 2) Support Vector Machine (SVM); 3) Decision Trees (DT); and 4) Ensemble Learning (EL).

#### 2.3.1 K-Nearest neighbors (KNN)

It is well known that the K-Nearest Neighbors algorithm (KNN) for machine learning (ML) has been introduced as the non-parametric approach employed to classify and regress areas [43]. Within these two areas, the input data includes k-closest training samples in the feature space. Therefore, output based on if the KNN is utilized to classify or regress is categorized into two parts as below:

1. In k-NN classification, the output is a class membership. An object is classified by a plurality vote of its neighbors, with the object being assigned to the class most common among its k nearest neighbors (k is a positive integer, typically small). If k = 1, then the object is simply assigned to that single nearest neighbor’s class.
2. In k-NN regression, the output is the property value for the object. This value is the average of the values of k nearest neighbors.

Moreover, according to the KNN classification, the output is a class membership. An object is categorized by a plurality vote of its neighbors so that the object has been allocated to the class that is the commonest among its k-nearest neighbors (k represents a positive integer, usually small). Therefore, if k = 1, then the object is readily allocated to the class of that single-nearest neighbor. Besides, KNN has been presented as one of the types of sample-based learning or lazy learning wherein the function is merely estimated locally, and each computation would be deferred till classification" [43].

#### 2.3.2 Support vector machine (SVM)

Vapnik and Cortes (1995) introduced SVM theory in which a hyperplane or a series of hyperplanes is constructed and utilized for both classification and regression [44,45]. By considering the labeled training set S = (*x*_*l*_, *y*_*l*_); l = 1;…; L of size L, and *y*_*l*_ ∈ 1,-1. The SVM can be obtained by solving:

$$\underset{w,b,\xi }{\text{min}}\frac{1}{2}w^{T}w+\sum_{i=l}^{L}\xi_{l}$$ (1)

subject to

$$y_{i}(w^{T}\phi (x_{l})+b)\geq 1-\xi_{l}$$ (2)

$$\xi_{l}\geq 0,l=1,\ldots ,L$$ (3)

where *ϕ*(*x*_*l*_) represents a nonlinear transformation mapping *x*_*l*_ in a high dimensional space that is called kernel function. The slack variable *ξ*_*l*_ represents nonlinearly separable training sets, and *C* denotes the parameter of a tunable positive regularization. To achieve a distributed SVM, (1) can be rewritten as follows:

$$\underset{w_{i},b_{i},\xi_{i}}{\text{min}}\frac{1}{2}\sum_{i=1}^{N}w_{i}^{T}w_{i}+C\sum_{i=1}^{N}\sum_{l=l}^{L}\xi_{il}$$ (4)

subject to

$$y_{il}(w_{i}^{T}\phi (x_{il})+b_{i})\geq 1-\xi_{il}$$ (5)

$$\xi_{il}\geq 0,i=1,\ldots ,N,l=1,\ldots ,L$$ (6)

where *N* denotes the number of groups working together to train the SVM and *w*_*i*_ represents the parameter of the local optimization for each group. By introducing a global variable *z*, the (4) can be reformulated as:

$$\underset{z,w_{i},b_{i},\xi_{i}}{\text{min}}\;\frac{1}{2}\sum_{i=1}^{N}\;z^{T}z_{i}+C\sum_{i=1}^{N}\;\sum_{l=l}^{L}\;\xi_{il}$$ (7)

$$y_{il}(w_{i}^{T}\phi (x_{il})+b_{i})\geq 1-\xi_{il}$$ (8)

$$\xi_{il}\geq 0,z=w_{i}$$ (9)

i=1,…,N,l=1,…,L (10)

To solve (7) distributively, variables {*z*, *w*_*i*_} *i* = 1,…, N can be partitioned into two sets represented by {*z*} and {*w*_*i*_}, *i* = 1,… N, and the Alternating Direction Method of Multipliers (ADMM) can be applied to solve the problem. Specifically, the scaled augmented Lagrangian function can be expressed as follows:

$$l\{z,w_{i},\xi_{i},\rho ,\mu_{i}\}=\frac{1}{2}\sum_{i=1}^{N}\;z^{T}z_{i}+C\sum_{i=1}^{N}\;\sum_{l=l}^{L}\;\xi_{il}+\frac{\rho }{2}∥w_{i}-z+\mu_{i}{∥}_{2}^{2}$$ (11)

where *ρ* denotes the step size and *μ*_*i*_ represents the scaled dual variable. At each iteration k, {*w*_*i*_},{z}and *μ*_*i*_ can be updated as follows:

$$w_{i}[k+1]=arg\underset{w_{i},b_{i},\xi_{i}}{\text{min}}C\sum_{i=1}^{L}\;\xi_{il}+\frac{\rho }{2}∥w_{i}-z[k]+\mu_{i}[k]{∥}_{2}^{2}$$ (12)

$$y_{il}(w_{i}^{T}\phi (x_{il})+b_{i})\geq 1-\xi_{il}$$ (13)

$$\xi_{ii}\geq 0,l=1,\ldots ,L.$$ (14)

Note that the process of updating the *w*_*i*_ can be done locally in the *i*_*th*_ group. Moreover, it involves the fitting of a *SVM* to the local data using an offset in the quadratic regularization term. The vector {z} is expressed as:

$$k_{i}[k+1]=arg\underset{z}{\text{min}}\frac{1}{2}z^{T}z+\frac{\rho }{2}∥w_{i}[k+1]-z+\mu_{i}[k]{∥}_{2}^{2}$$ (15)

which can be solved analytically as:

$$z=\frac{N_{\rho }}{\frac{1}{\rho }+N_{\rho }}(\overset{-}{w}[k+1]+\overset{-}{\mu }[k])$$ (16)

in which $\overset{-}{w}=\frac{1}{N}\sum_{i=1}^{N}\;w_{i}$ and $\overset{-}{\mu }=\frac{1}{N}\sum_{i=1}^{N}\;\mu_{i}$. Finally, the scaled dual variable *μ*_*i*_ can be updated by:

$$\mu_{i}[k+1]=\mu_{i}[k]+w_{i}[k+1]-z[k+1]$$ (17)

In constructing historical data, since the number of classes in topology samples depends on the number of switches, so the number of classes can be more than two groups, therefore a multi-class SVM is used [46,47], and for each nonlinear classifier, Gaussian kernel is applied in which *δ* is a mutable parameter. The main factors affecting the performance of SVM comprise kernel function and its parameters as well as the soft margin parameter C. The optimal Gaussian kernel parameter and soft margin (C) can be used for improving the efficiency of nonlinear SVM. Gaussian kernel, which has a single parameter (*γ*), is a typical choice for SVM [48]. The common practice for finding the best values of C and *γ* is to conduct a grid search, i.e., repeat the calculations with different C and *γ* combinations and determine the values yielding the best accuracy through cross-validation [49]. In Step 1, historical data are prepared by the independent system operator (ISO). In Step 2, the local and global optimization parameters are initialized. Step 3 comprises two parts. First, the local optimization parameter *w*_*i*_ is obtained by solving the optimization problem defined in (12) under constraints (13) and (14). Then, the global optimization parameter *z* is optimized in (16), where $\overset{-}{w}$ and $\overset{-}{\mu }$ are obtained by in-network processing through messages that were only exchanged among neighboring groups. Following the optimization solution and the achievement of convergence of *w*_*i*_ by each group, the local and global optimization parameters, i.e., *w*_*i*_ and *z*, respectively, are returned in Step 4.

#### 2.3.3 Decision Tree (DT)

A Decision Tree (DT) is a predictive model where supervised learning and non-parametric methods with a hierarchical structure are used to classify different data types, and results are delivered in a flowchart with a tree-like structure. According to the dependent variable type, this algorithm is divided into two categories: regression trees for continuous variables and classification trees for discrete variables. In the DT algorithm, fragmentation of data is implemented using the features as a tree, and for better understanding, it is written using if-then rules [50]. Depending on the training data, a feature for the data is selected at each stage of this method, and the data set is decomposed into a further class grouping based on chosen features. This process continues until all the data in a category has a single label.

#### 2.3.4 Ensemble Learning (EL)

Ensemble Learning (EL) methods represent a machine learning approach in which multiple learning algorithms are used in a parallel fashion to achieve more acceptable predictive functions in comparison to the situation that may be obtainable from each constituent learning algorithm [51,52]. While in statistical mechanics, a statistical ensemble is generally infinite, an ML ensemble contains a finite set of alternative models. This, in turn, leads to a structure with higher flexibility than the individual alternatives. Ensemble methods are meta-algorithms that combine several machine learning techniques into one predictive model to decrease variance (bagging), bias (boosting), or correlation (random subspace).

#### 2.3.5 Bagging algorithm

Bagging, also known as bootstrap aggregation, is a machine learning ensemble meta-algorithm designed to improve machine learning algorithms’ stability and accuracy in statistical regression and classification. Moreover, the algorithm can decrease the variance and prevent overfitting. Leo Breiman (1994) proposed bagging to improve the classification by combining classifications of randomly produced training sets. The bagging method is modeled based on the instability of base learners, which can be utilized to modify such unstable base learners’ predictive performance. The main idea is that, given a training set *S* of size *n* and a learner *L*, which commonly is a decision tree, bagging creates *m* new training sets *S*_*i*_ with replacement. Then, the bagging algorithm applies *L* to each *S*_*i*_ to build *m* models. The final output of bagging is based on simple averaging [53].

Even though it is commonly used in combination with DT methods, bagging can be applied with any kind of technique. In fact, bagging is a special case for the model averaging approach [52].

#### 2.3.6 Boosting algorithm

In ML, boosting is an ensemble meta-algorithm designed primarily to reduce bias [54], and variance in supervised learning includes a family of ML algorithms that convert weak learners into strong ones [55]. Boosting is based on the question first posed by [56,57]: "Can a set of weak learners create a single strong learner?" A weak learner is defined to be a classifier that is only slightly correlated with the proper classification (although it can label examples better than random guessing). In contrast, a strong learner is a classifier that is arbitrarily well-correlated with the true classification. While boosting is not algorithmically constrained, most boosting algorithms consist of learning weak classifiers concerning distribution and adding a final strong classifier. After the addition, the weak learners are weighted in a way related to the weak learners’ accuracy, and the data weights are readjusted, a process is known as “re-weighting.” Misclassified input data gain a higher weight while examples that are classified correctly lose weight. Thus, future weak learners focus more on the examples than on previous misclassified weak learners.

#### 2.3.7 Random subspace algorithm

**Fig 1:**
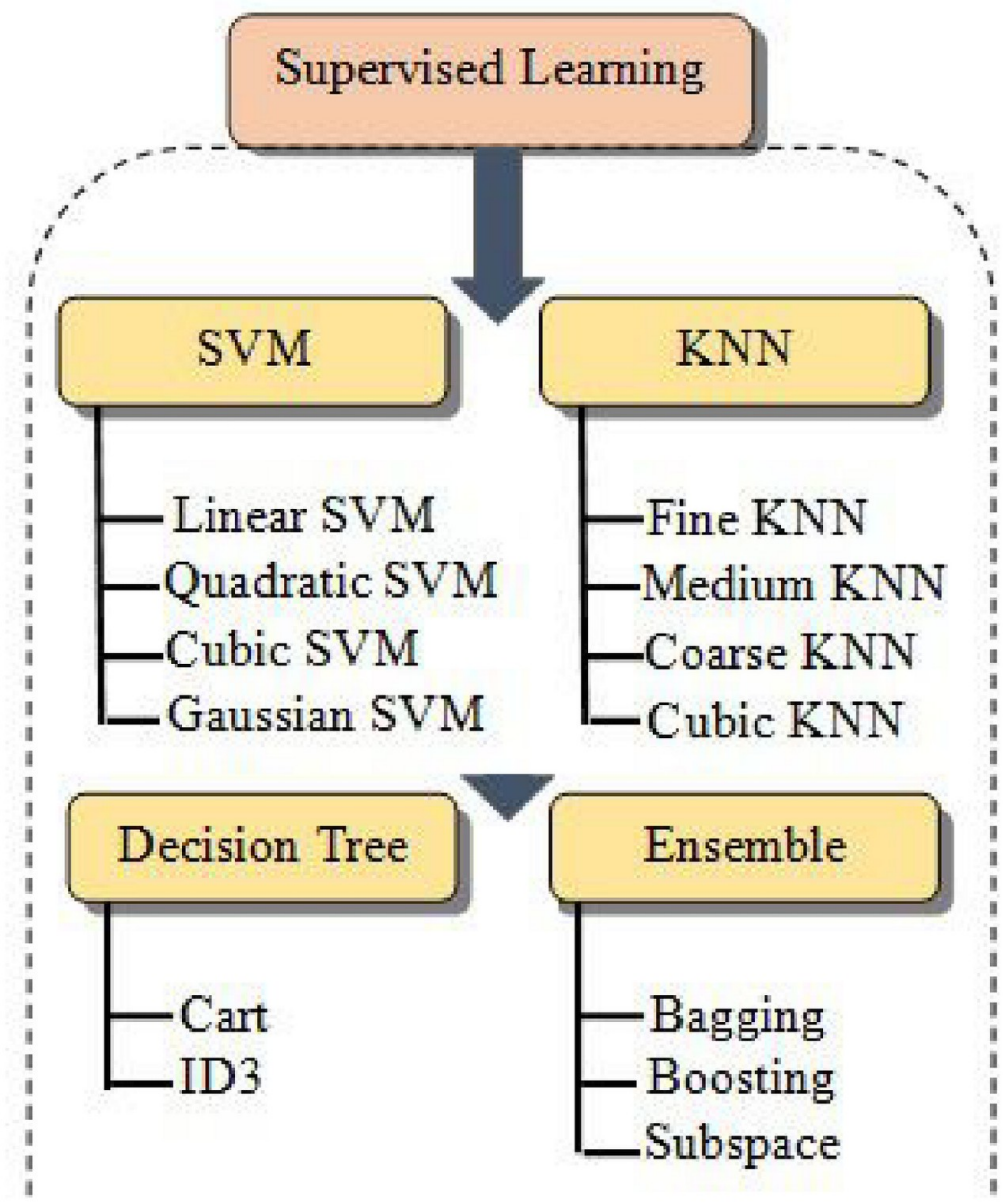
Classification of SML algorithms. These methods are in a supervised paradigm.

In ML, the random subspace method, also called attribute bagging or feature bagging, is an ensemble learning method that attempts to reduce the correlation between estimators in an ensemble by training them on random samples of features instead of the entire feature set. The random subspace method is similar to bagging, except that the features are randomly sampled with the replacement for each learner. Informally, this prevents individual learners from over-focusing on features that appear highly predictive/descriptive in the training set but fail to be as predictive for points outside that set. For this reason, random subspace algorithms are an attractive choice for problems where the number of features is much larger than the number of training points. The random subspace method has been used with DT. When combined with "ordinary" bagging of decision trees, the resulting models are called random forests. The method has also been applied to linear classifiers, support vector machines, k-nearest neighbors, and other types of classifiers [58]. Fig 1 summarizes the main classification of SML.

### 2.4 Dataset

#### 2.4.1 Description of the dataset used to test the method

We consider 7 switch devices in simulation, implying 7 states for possible events, and correspondingly, 7 voltage curves in the related time domain for 15 seconds (the simulation time duration). We set the voltage changes per second as a first feature. Maximum and minimum of the instantaneous voltage are considered as second and third features. Hence, the first column of Excel files in the supplementary material describes when the event occurs. Voltage changes, its maximum, and minimum are featured in the second, third, and fourth columns. Each excel file indicates one state of event or fault, including transient voltage and type of fault. The excel file is available at: https://doi.org/10.6084/m9.figshare.14123018.v2.

#### 2.4.2 Rationale for choosing a dataset

The power system analysis is generally done by monitoring of current status and computing several distinct parameters. One of the most important parameters in the power system is the transient voltage. As is well known, any change in the network, such as line and generation outages, affects the network operation status and its parameters, such as voltage in transient and steady-state mode. Thus, the tracking of the status of the current power system requires the analysis of the transient voltage. To simulate the fault in the network, we used the switch, so that when a switch is disconnected, a line or generation unit is disconnected from the network. This in turn implies that the analysis of the power system where the fault occurred requires tracking the changes in the network transient voltage. The changes and faults that occurred in the network are exactly proportional to the voltage changes, which means that the machine learning process can learn all the voltage changes in all possible fault scenarios and, after the training process, can detect the type of fault and the fault scenario that occurred.

#### 2.4.3 The source of the data and how it was obtained

We simulated a real standard power distribution system in ETAP software. To simulate the fault, we used 7 power switches, and, consequently, any switch disconnecting is considered a network fault. We simulated the fault in the network and recorded the transient voltage after the fault. For each fault, we obtained a transient voltage from the power grid. Hence, the labels define the type of fault, while the output is represented by the transient voltage. We recorded a series of transient voltages for all possible faults until we could train the machine learning process using these transient voltages and types of faults.

## 3 Results and discussion

### 3.1 Tracking the switching devices

**Fig 2:**
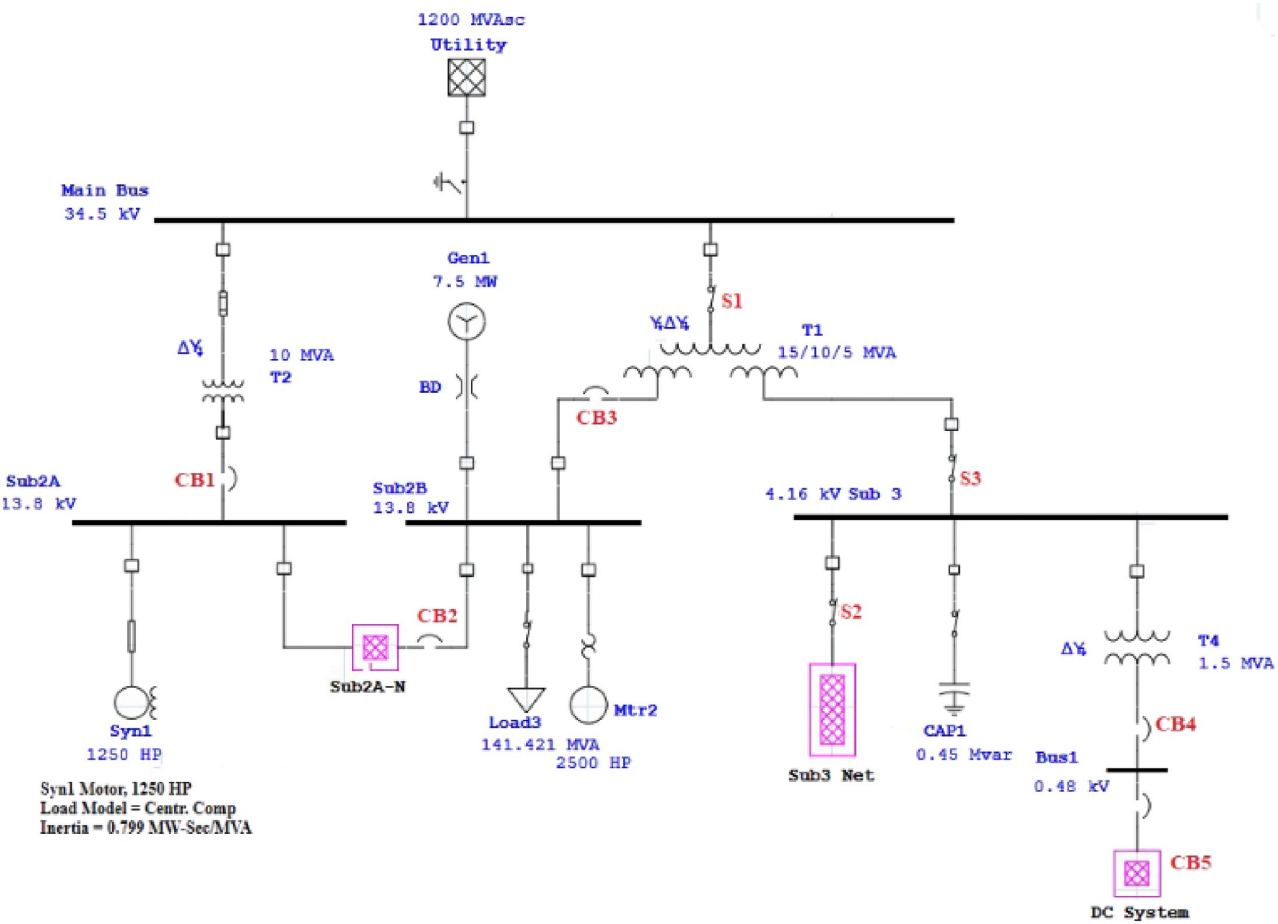
An ANSI case study. This case study is considered a distribution network.

**Fig 3:**
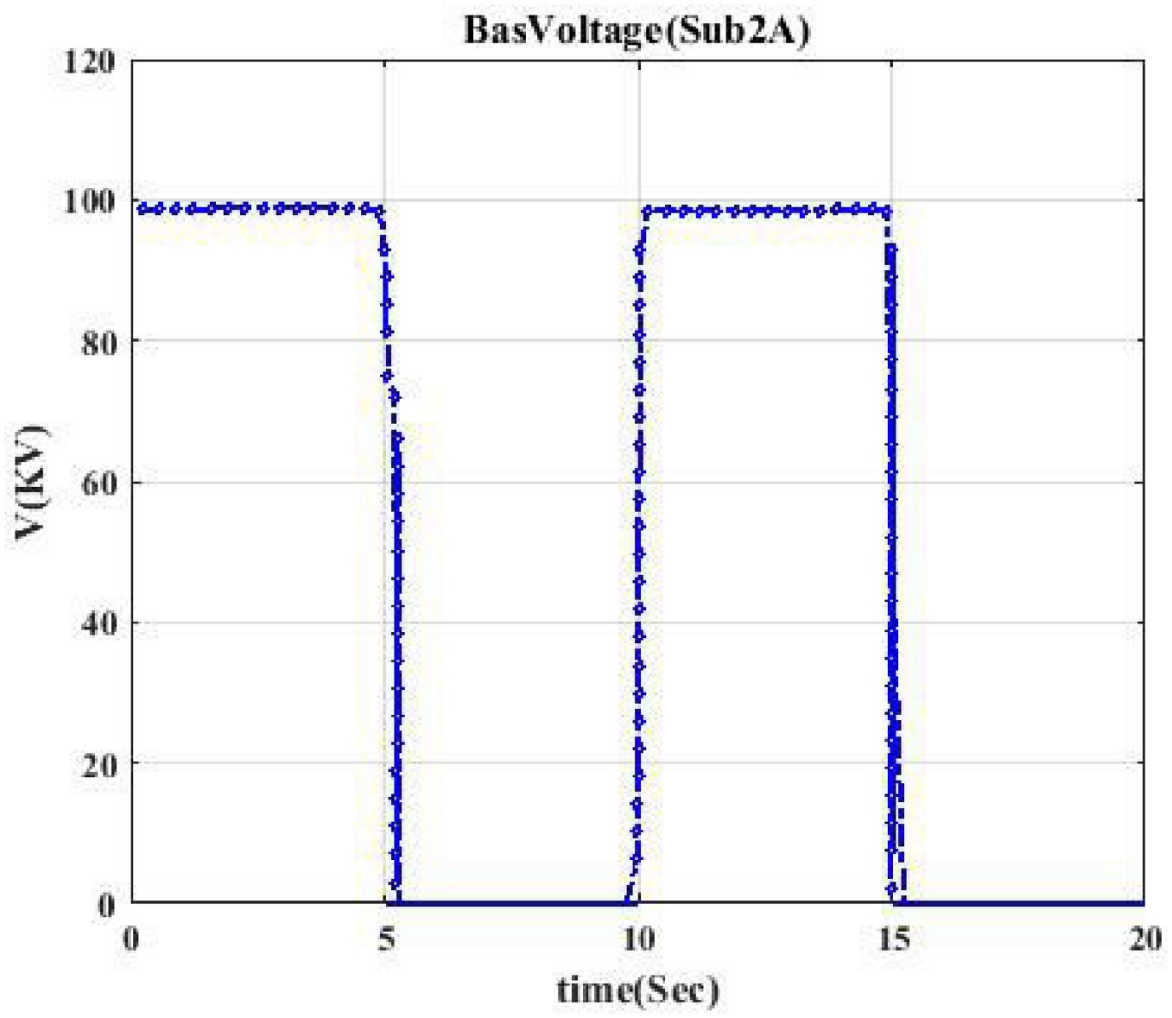
Voltage profile related to switching Sub2A-CB1. This shows the voltage curve when circuit breaker CB1 is switched.

**Fig 4:**
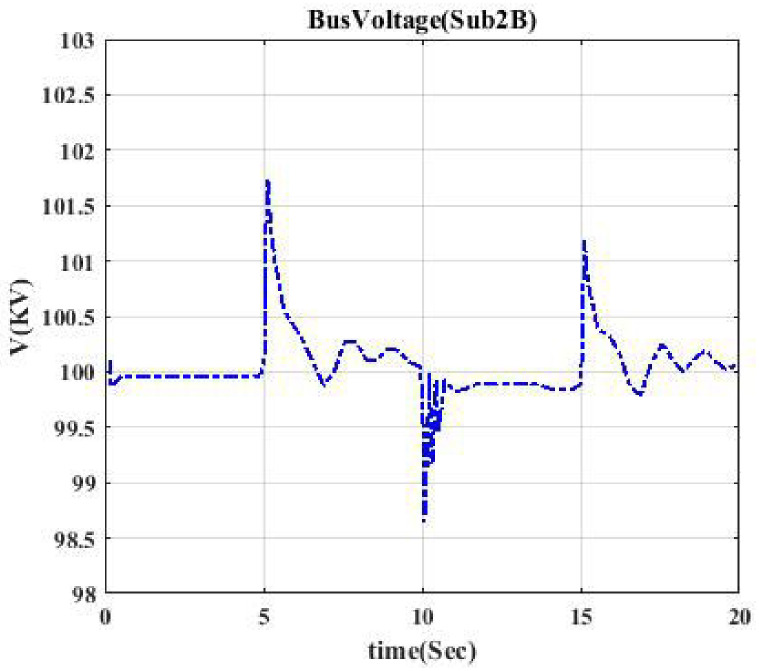
Voltage profile related to switching Sub2B-CB3. This shows the voltage curve when circuit breaker CB3 is switched.

**Fig 5:**
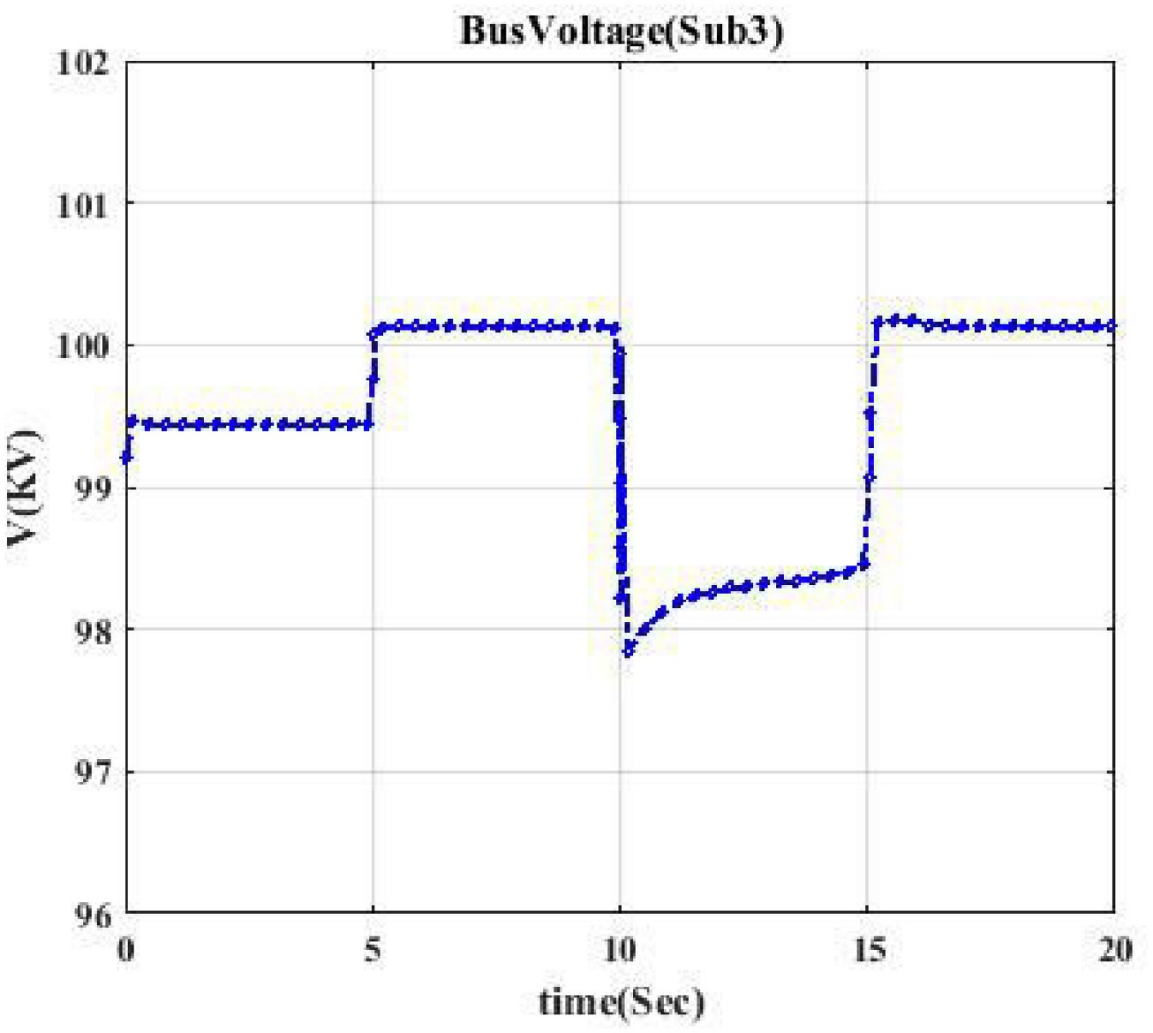
Voltage profile related to switching Sub3-S2. This shows the voltage curve when switch S2 is switched.

**Fig 6:**
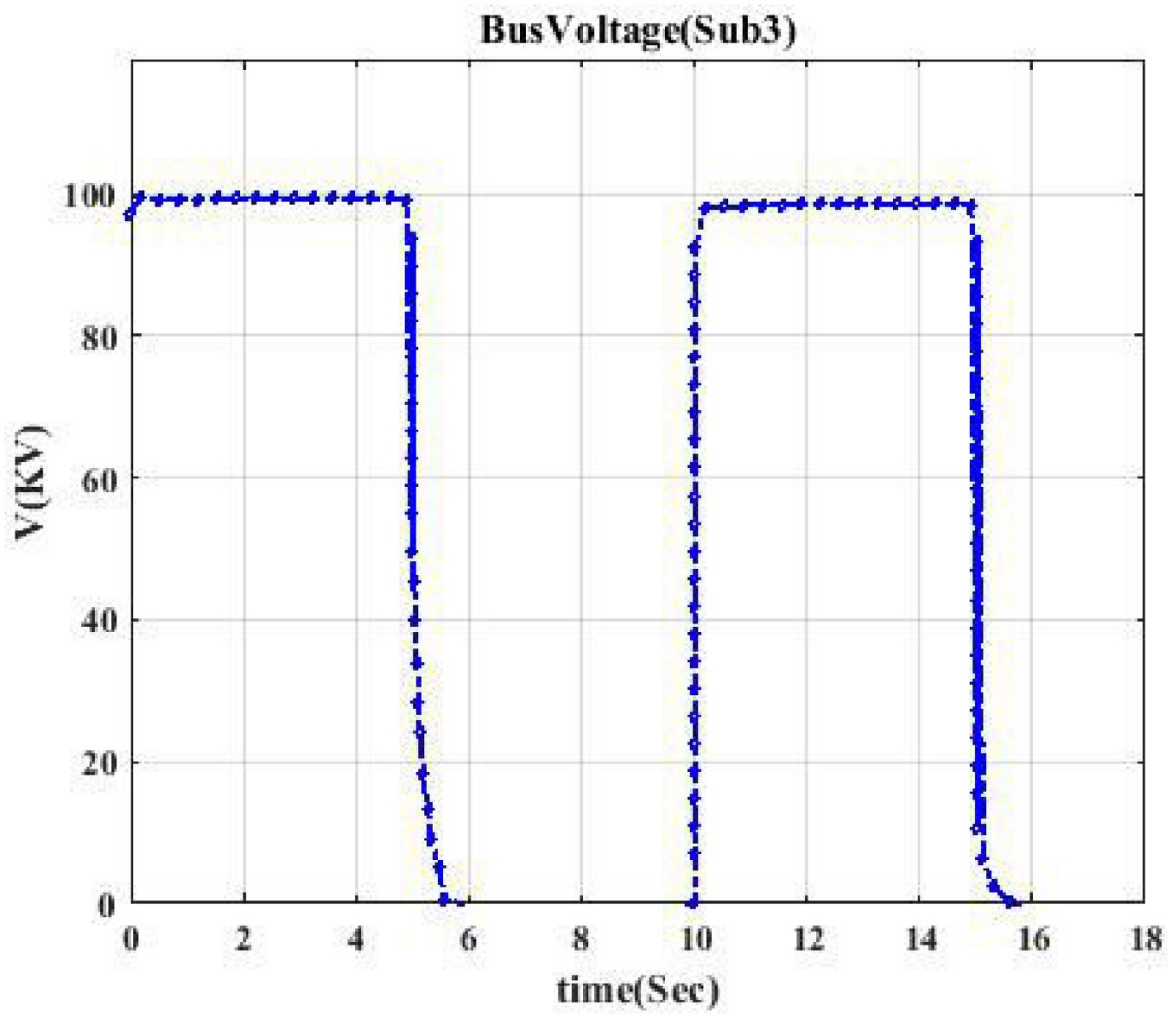
Voltage profile related to switching Sub3-S3. This shows the voltage curve when switch S3 is switched.

**Fig 7:**
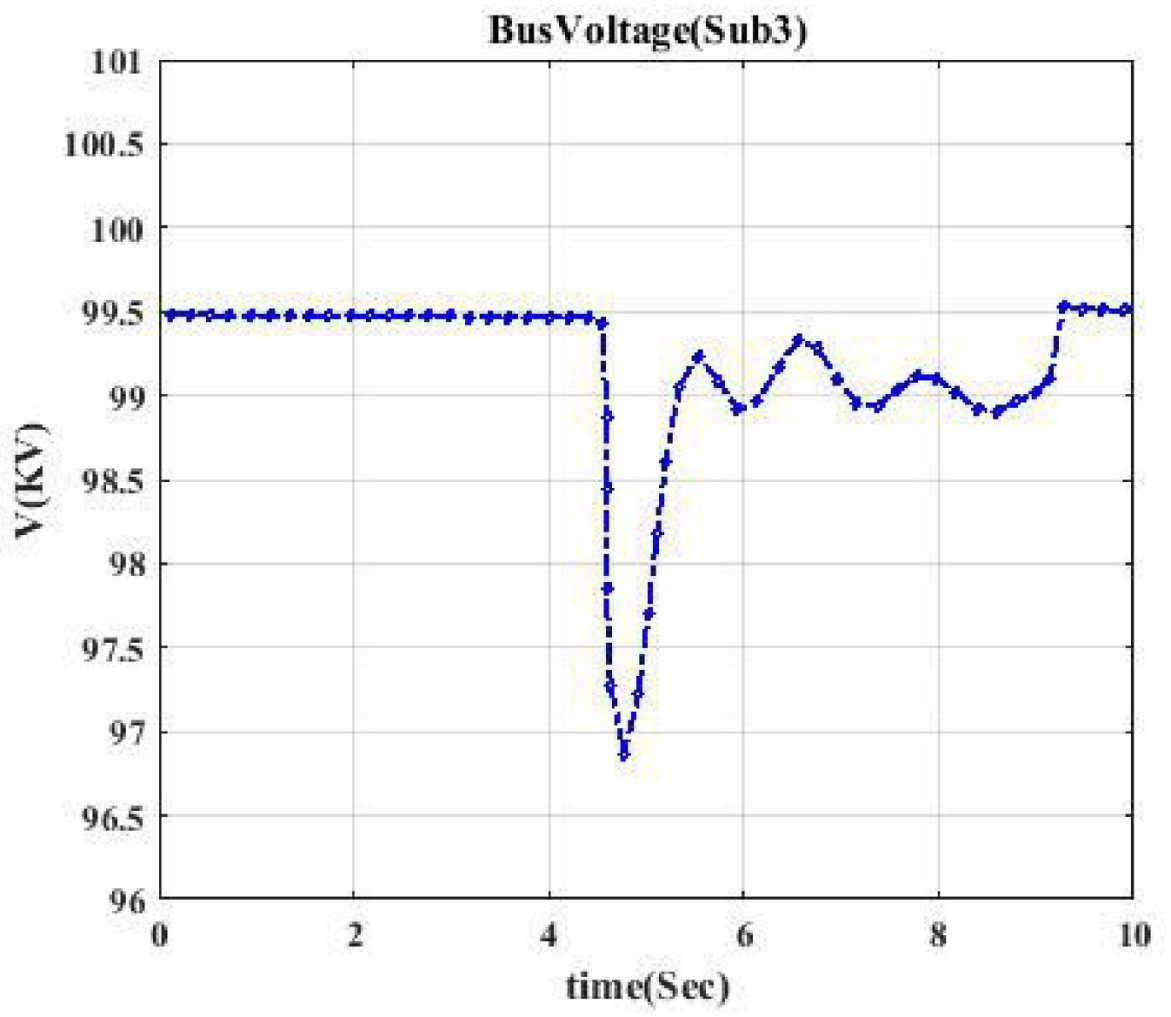
Voltage profile related to switching Sub3-S1. This shows the voltage curve when switch S1 is switched.

First, we analyze each switching device’s status, such as the circuit breaker and its impact on the main feeder’s voltage profile. Fig 2 shows an ANSI case study as an example distribution network, including the main bus as a utility and three sub-bus and several AC and DC sub-network. In this case study, we consider seven switching devices such as, i.e., the circuit breaker, which can be seen in red color in Fig 2. Thus, we apply 7-class supervised learning. We define an event for each device separately and analyze the transient stability of the system in ETAP software. We assume all devices in t = 5 are opened, in t = ten are closed, and in t = 15 are opened again separately. For some switching devices, voltage profiles in different buses were analyzed. Figs 3–7 show the voltage profiles related to each switching event. According to these graphs, each voltage profile characteristic at the switching time is more important than the switching time since each circuit breaker or other switching devices may be switched several times over the same period of time.

Moreover, the voltage profile related to each typical switching event displays intermittent nature. According to each switching event’s voltage profile, we categorize them into seven classes to model a trainable machine. Therefore, our model categorizes events into 7 clusters since we have seven switching devices, resulting in a trainable machine as a 7-class machine. Fig 3 indicates the voltage profile in Bus Sub2A when circuit breaker CB1 is switched. According to Fig 2, after switching CB1, Bus A (Sub2A) is disconnected from the main bus, and only one transformer goes out of the circuit. According to Fig 3, the voltage drop is like a pulse.

Figs 4 and 5 indicate the voltage profile in Bus Sub2B and Bus Sub3 when circuit breaker CB3 and switch S2 are switched, respectively. According to Fig 2, after switching CB3, Bus 2 (Sub2B) is disconnected from Bus 3, and not only one transformer goes out of the circuit, but also the switching of the DC source affects Bus 2B since the voltage profile in Bus 2B looks like a parabola as Fig 4. Fig 5 shows a similar situation. Figs 6 and 7 show the voltage profile in Bus3 when switching S3 and S1 are switched, respectively.

**Fig 8:**
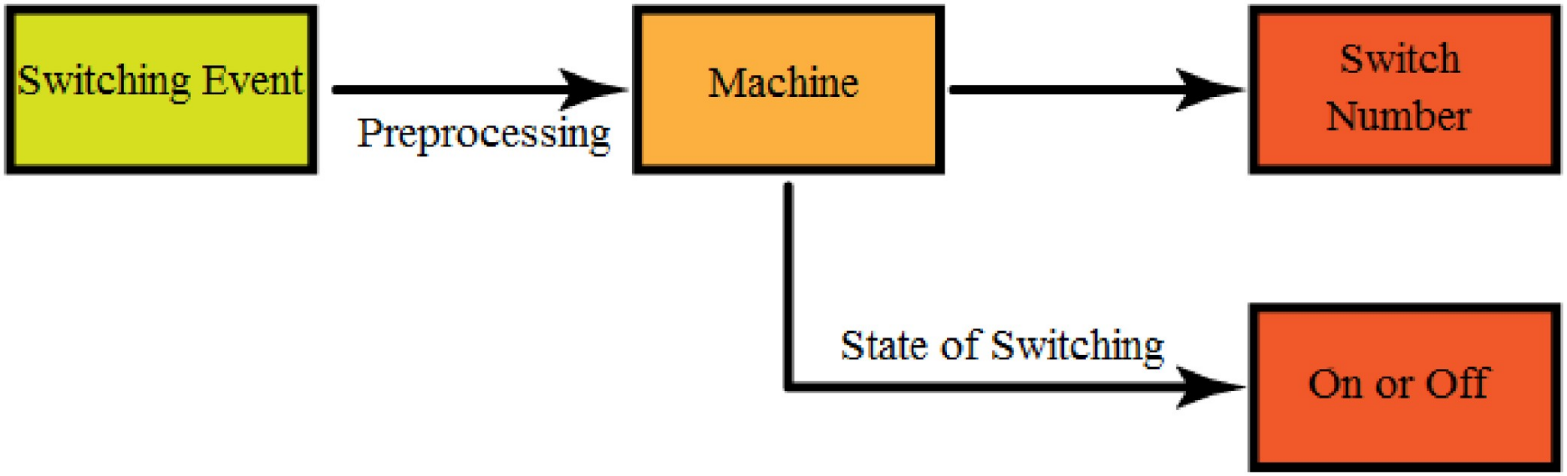
Machine Learning (ML) process. The methodology of topology identification.

In summary, using the supervised paradigm, we can detect which circuit breaker or other switching devices are switched, and we can identify which switch has been connected or disconnected. Fig 8 displays the topology identification (TI) process and its performance of the two tasks: 1) detection of switch number and 2) detection of the state of switching.

Tables 1–3 show the KNN, SVM, and EL algorithms’ performance, respectively, in the test process and after the training process. In the test, the process machine is evaluated by the samples which have not been observed before. The accuracy of the learning algorithm is commonly evaluated according to the following formula:

$$\text{Accuracy}\;(\text{in}\;\text{percent})=(\frac{\text{correct}\;\text{predicted}\;\text{samples}}{\text{all}\;\text{samples}})\times 100$$ (18)

**Table 1:** Percentage error of alternative KNN algorithms.

Method	Accuracy	Time (in seconds)
**Fine KNN**	98.2%	5s
**Medium KNN**	97.6%	4s
**Cubic KNN**	96.7%	9s
**Coarse KNN**	90.2%	10s

**Table 2:** Percentage error of alternative SVM algorithms.

Method	Accuracy	Time (in seconds)
**Linear SVM**	100%	8s
**Quadratic SVM**	100%	10s
**Cubic SVM**	100%	9s
**Gaussian SVM**	100%	13s

**Table 3:** Percentage error of alternative Ensemble algorithms.

Method	Accuracy	Time (in seconds)
**Boosting**	21%	10s
**Bagging**	95.8%	11s
**Subspace KNN**	94.5%	13s
**RUS Boosted Tree**	21%	15s

The more the machine detects the correct samples, the more accurate it will be. The results clearly indicate that supervised algorithms can identify switches’ status and the system’s topology with a significant robustness degree. According to Tables 1 and 2, all of the KNN and SVM algorithms display a proper performance and can be viewed as a suitable algorithm for topology identification with high accuracy and low time duration. According to Table 3, the EL algorithms (bagging and random subspace KNN) also display a high performance. However, the accuracy metric for boosting and RUS boosted tree is not as good as expected.

**Fig 9:**
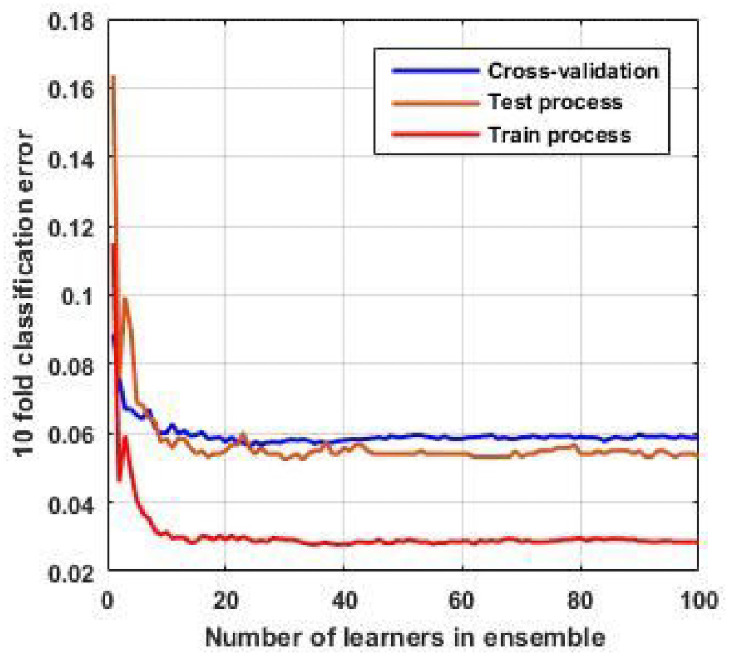
Error classification for KNN random subspace method. This curve indicates the performance of the algorithm.

Fig 9 shows the classification error of 10-fold cross-validation for EL with the KNN subspace method. As we see, by selecting the number of learners equal to 100, we have proper accuracy in our model. According to Fig 9, the error rate is 5.5% in the test process, suggesting that we can detect any switching event and identify the distribution network’s topologies with a satisfactory degree of accuracy.

### 3.2 Tracking of the special load switch

**Fig 10:**
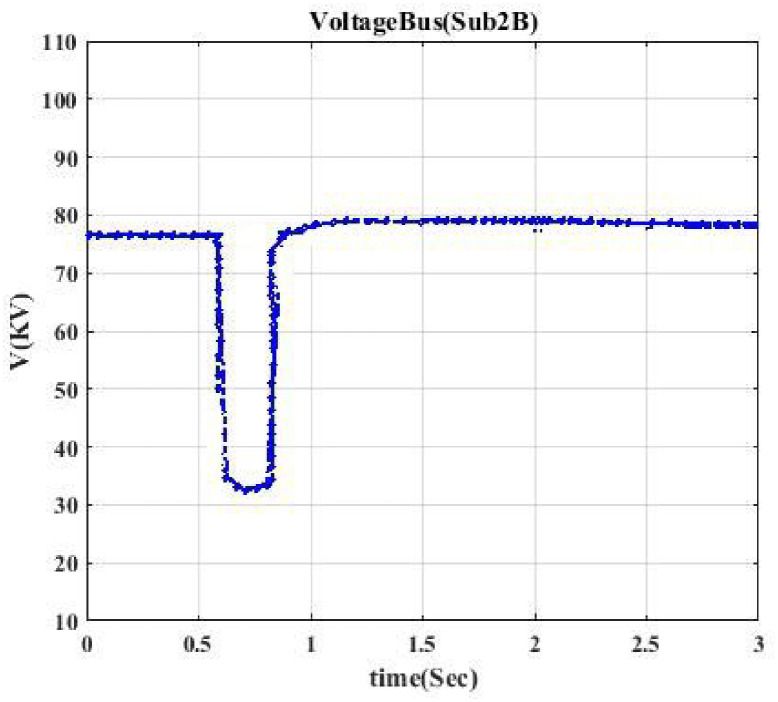
Voltage profile for switching the static load. This curve indicates the voltage curve when the static load is disconnected from the system.

**Fig 11:**
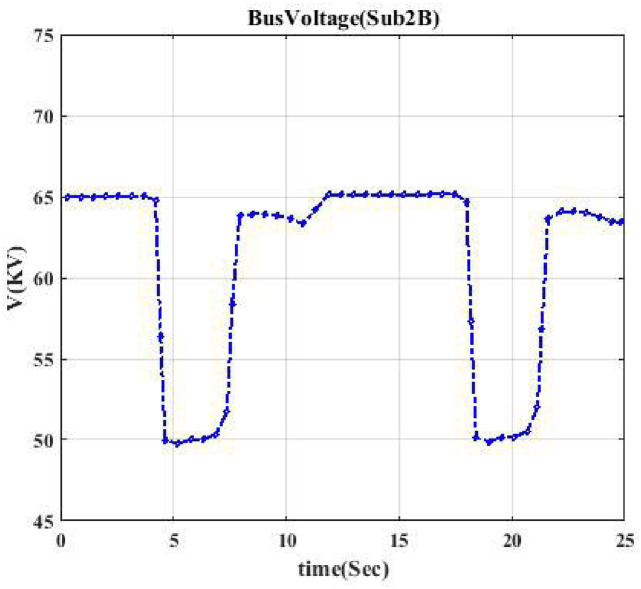
Voltage profile for switching the induction motor. This curve indicates the voltage curve when the induction motor is disconnected from the system.

**Fig 12:**
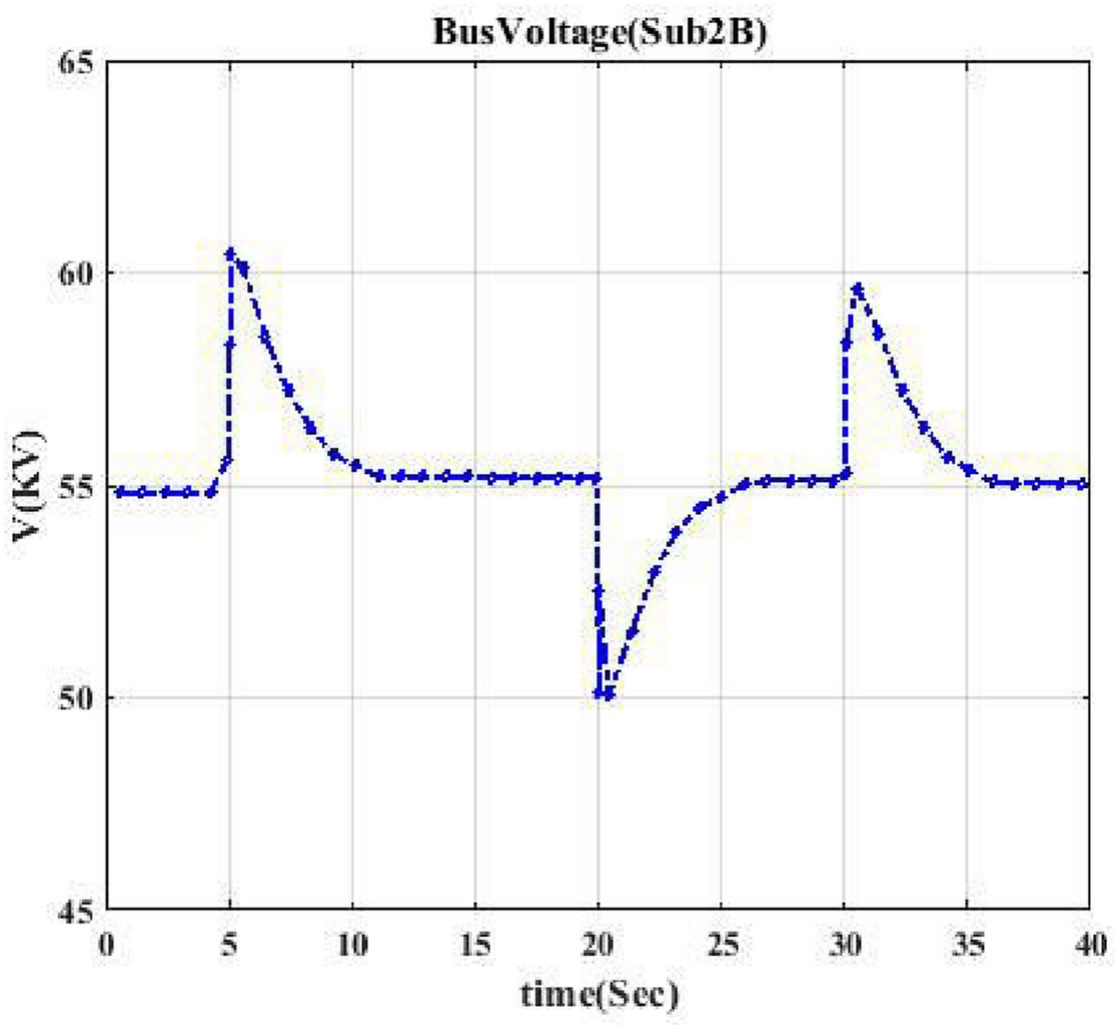
Voltage profile for switching the electric vehicle. This curve indicates the voltage curve when the electric vehicle is disconnected from the system.

It is possible that after any switching event, several loads are disconnected from the system at the same time. In this section, we show that our approach is capable of detecting switching events when only one load is connected to the switch. We assume several switches even though only one type of special load is connected to these switches, e.g., after switching event, only one of them is connected or disconnected to the feeder. We consider three different types of loads, i.e., induction motor, static load, and electric vehicle. The main goal is to categorize the voltage profile related to these three different types of loads. Figs 10–12 show voltage profiles related to these switching events. Fig 10 indicates the voltage profile when the event is on the static load. In other words, when the switch for the static load is disconnected or connected from the rest of the grid.

**Fig 13:**
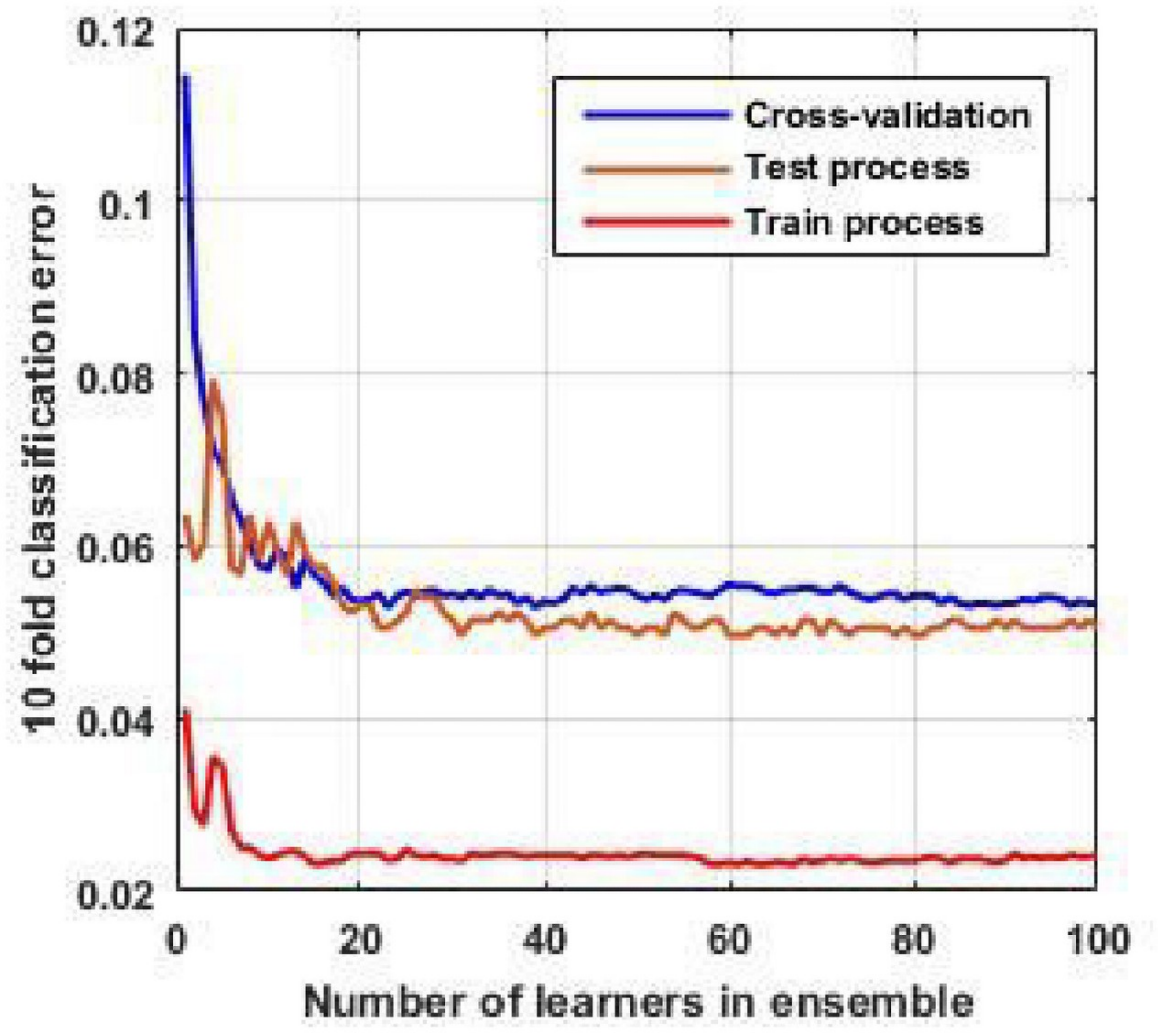
Error classification for KNN random subspace method. This curve indicates the performance of the algorithm.

Fig 11 indicates the voltage profile when the event is on the induction motor, resulting in being disconnected from the grid twice. Fig 12 shows the voltage curve, a parabola, indicating that a DC source has been disconnected from the network. Table 4 shows the accuracy results. We conclude that the proposed methodology can accurately detect the type of load connected or disconnected to the distribution system. Fig 13 shows the percentage error of ensemble learning with the KNN random subspace method. The model has a satisfactory topology identification (TI) performance by selecting the number of learners equal to 100. As one can observe, the error is below 6 percent.

**Table 4:** Percentage error of alternative SVM algorithms.

Method	Accuracy	Time (in seconds)
**Quadratic SVM**	100%	10s
**Subspace KNN**	95%%	15s
**Cubic KNN**	100%	14s
**Bagging**	100%	13s
**Gaussian SVM**	100%	16s

According to Fig 13, the test process presents an error rate of 5 percent. This result suggests that we can detect any switching event on various loads and track the special load switch.

## 4 Conclusions

This paper proposes an approach to topology identification (TI) of the distribution system based on machine learning algorithms. This methodology does not require any sensors and measurement devices to analyze the feeder’s voltage profile. This stresses this point since switching devices’ changes affect the feeder’s voltage profile in the distribution network. We show that by tracking the voltage profile’s behavior in each feeder with an ML algorithm, we can detect switching devices’ status and identify the distribution system’s typologies. It should be noted that the concept of voltage used in the paper is confined to transient voltage. That is, the transient voltage generated by an event in the network is completely different the one generated by another event. Alternatively, the voltage across the network is dependent upon different events. The transient voltage is similar to a label for a specific event, which detects different switching modes by examining the transient voltage and in final analysis diagnosing the network topology. However, in the case where the voltage is in steady-state mode, the identification from only the voltage curves cannot solve the non-radiative grid topology. We also show that by tracking the status of switching devices, the ML approach can detect a high degree of accuracy, which kind of load is connected or disconnected to the system.

## References

[1] Bernards R. Smart planning: integration of statistical and stochastic methods in distribution network planning. 2018.

[2] Zhou Q, Bialek JW. Generation curtailment to manage voltage constraints in distribution networks. IET Generation, Transmission & Distribution. 2007 5 1;1(3):492–8.

[3] Clement-Nyns K, Haesen E, Driesen J. The impact of charging plug-in hybrid electric vehicles on a residential distribution grid. IEEE Transactions on power systems. 2009 12 18;25(1):371–80.

[4] Lopes JA, Soares FJ, Almeida PM. Integration of electric vehicles in the electric power system. Proceedings of the IEEE. 2010 10 4;99(1):168–83.

[5] Sharon Y, Annaswamy AM, Motto AL, Chakraborty A. Topology identification in distribution network with limited measurements. IEEE PES Innovative Smart Grid Technologies (ISGT) 2012 1 16: 1–6.

[6] Singh R, Manitsas E, Pal BC, Strbac G. A recursive Bayesian approach for identification of network configuration changes in distribution system state estimation. IEEE Transactions on Power Systems. 2010 3 4;25(3):1329–36.

[7] Singh N, Glavitsch H. Detection and identification of topological errors in online power system analysis. IEEE Transactions on Power Systems. 1991 2;6(1):324–31.

[8] Singh N, Oesch F. Practical experience with rule-based online topology error detection. IEEE Transactions on power systems. 1994 5;9(2):841–7.

[9] Wang J, Ge M, Sun H, LI Q, WU W, GUO Q, et al. A comprehensive rule-based method for substation topology error identification. Power System Technology. 2012 5;36(5):166–72.

[10] Souza JC, Da Silva AL, de Silva AA. Online topology determination and bad data suppression in power system operation using artificial neural networks. IEEE Transactions on Power Systems. 1998 8;13(3):796–803.

[11] Gao Y, Zhang Z, Wu W, Liang H. A method for the topology identification of distribution system. In2013 IEEE Power & Energy Society General Meeting 2013 7 21 (pp. 1–5). IEEE.

[12] Clements KA, Costa AS. Topology error identification using normalized Lagrange multipliers. IEEE Transactions on power systems. 1998 5;13(2):347–53.

[13] Chen Y, Zhou J, Yu E, Li Q, Wang L. Transfer power flow approach to topology error identification. Automation of Electric Power Systems. 2010 1;34(1):20–4.

[14] Chen Y, He G, Zhou J, Yu E, Li Q, Gu Z, et al. An improved power flow transfer approach with enhanced ability to identify topology error and bad data. Power System Technology. 2012;36(3):95–100.

[15] Clements KA, Davis PW. Detection and identification of topology errors in electric power systems. IEEE Transactions on Power Systems. 1988 11;3(4):1748–53.

[16] Lugtu RL, Hackett DF, Liu KC, Might DD. Power system state estimation: Detection of topological errors. IEEE Transactions on Power Apparatus and Systems. 1980 11 (6):2406–12.

[17] Dorostkar-Ghamsari MR, Fotuhi-Firuzabad M, Lehtonen M, Safdarian A. Value of distribution network reconfiguration in presence of renewable energy resources. IEEE Transactions on Power Systems. 2015 8 5;31(3):1879–88.

[18] Bolognani S, Bof N, Michelotti D, Muraro R, Schenato L. Identification of power distribution network topology via voltage correlation analysis. In52nd IEEE Conference on Decision and Control 2013 Dec 10 (pp. 1659–1664). IEEE.

[19] Xu S, De Lamare RC, Poor HV. Dynamic topology adaptation for distributed estimation in smart grids. In2013 5th IEEE International Workshop on Computational Advances in Multi-Sensor Adaptive Processing (CAMSAP) 2013 Dec 15 (pp. 420–423). IEEE.

[20] Huang J, Gupta V, Huang YF. Electric grid state estimators for distribution systems with microgrids. In2012 46th Annual Conference on Information Sciences and Systems (CISS) 2012 Mar 21 (pp. 1–6). IEEE.

[21] Jing M, Wuyu Z. Power network topological analysis based on incidence matrix notation method and loop matrix [J]. Automation of Electric Power Systems. 2014;38(12):74–80.

[22] Yao Y, Ye L, Wu Z, Wang D. Analysis of network topology by the matrix method with sparse matrix techniques [J]. Power System Protection and Control. 2011;39(5):31–4.

[23] Taylor JA, Hover FS. Convex models of distribution system reconfiguration. IEEE Transactions on Power Systems. 2012 2 16;27(3):1407–13.

[24] Lavorato M, Franco JF, Rider MJ, Romero R. Imposing radiality constraints in distribution system optimization problems. IEEE Transactions on Power Systems. 2011 8 11;27(1):172–80.

[25] Pappu SJ, Bhatt N, Pasumarthy R, Rajeswaran A. Identifying topology of power distribution networks based on smart meter data. IEEE Trans Smart Grid. https://doi. org/10.1109/TSG. 2017.

[26] Sun X, Lv D, Wang H, Hou M, Zhang Z. Topology identification method based on multi-agent for outage areas in multi-contact distribution network. In2016 IEEE International Conference on Power and Renewable Energy (ICPRE) 2016 Oct 21 (pp. 456–459). IEEE.

[27] Clements KA, Costa AS. Topology error identification using normalized Lagrange multipliers. IEEE Transactions on power systems. 1998 5;13(2):347–53.

[28] Mili L, Steeno G, Dobraca F, French D. A robust estimation method for topology error identification. IEEE Transactions on Power Systems. 1999 11;14(4):1469–76.

[29] Deka D, Backhaus S, Chertkov M. Structure learning and statistical estimation in distribution networks-part i. arXiv preprint arXiv:1501.04131. 2015 Jan 16.

[30] Cavraro G, Arghandeh R, von Meier A. Distribution network topology detection with time series measurement data analysis. arXiv preprint arXiv:1504.05926. 2015 Apr 22.

[31] Sharon Y, Annaswamy AM, Motto AL, Chakraborty A. Topology identification in distribution network with limited measurements. In2012 IEEE PES Innovative Smart Grid Technologies (ISGT) 2012 Jan 16 (pp. 1–6). IEEE.

[32] Korres GN, Manousakis NM. A state estimation algorithm for monitoring topology changes in distribution systems. In2012 IEEE Power and Energy Society General Meeting 2012 Jul 22 (pp. 1–8). IEEE.

[33] Baran ME, Jung J, McDermott TE. Topology error identification using branch current state estimation for distribution systems. In2009 Transmission & Distribution Conference & Exposition: Asia and Pacific 2009 Oct 26 (pp. 1–4). IEEE.

[34] Arghandeh R, Gahr M, von Meier A, Cavraro G, Ruh M, Andersson G. Topology detection in microgrids with micro-synchrophasors. In2015 IEEE Power & Energy Society General Meeting 2015 Jul 26 (pp. 1–5). IEEE.

[35] Zhang Q. Adaptive observer for multiple-input-multiple-output (MIMO) linear time-varying systems. IEEE transactions on automatic control. 2002 8 7;47(3):525–9.

[36] Farza M, M’Saad M, Maatoug T, Kamoun M. Adaptive observers for nonlinearly parameterized class of nonlinear systems. Automatica. 2009 10 1;45(10):2292–9.

[37] Nielsen MA. Neural networks and deep learning. San Francisco, CA: Determination press; 2015 9 25.

[38] Goodfellow I, Bengio Y, Courville A. Deep learning book. MIT Press. 2016 8;521(7553):800.

[39] Mehta P, Bukov M, Wang CH, Day AG, Richardson C, Fisher CK, et al. A high-bias, low-variance introduction to machine learning for physicists. Physics reports. 2019 5 30; 810:1–24. 10.1016/j.physrep.2019.03.001 31404441 PMC6688775

[40] Simon P. Too big to ignore: the business case for big data. John Wiley & Sons; 2013 3 18.

[41] Kohavi R. Glossary of terms. Special issue on applications of machine learning and the knowledge discovery process. 1998;30(271):127–32.

[42] Russell S, Norvig P, Canny JF, Malik J, Edwards DD, Artificial Intelligence: A Modern Approach. 2nd ed. Prentice Hall Series in Artificial Intelligence. Upper Saddle River. NJ: Prentice Hall/Pearson Education. 2003.

[43] Sutton RS, Barto AG. Reinforcement learning: An introduction. MIT press; 2018 10 19.

[44] Cortes C, Vapnik V. Support-vector networks. Machine learning. 1995 9 1;20(3):273–97.

[45] Vapnik V, Vapnik V. Statistical learning theory Wiley. New York. 1998; 1:624.

[46] Duan KB, Keerthi SS. Which is the best multi-class SVM method? An empirical study. In International workshop on multiple classifier systems 2005 6 13 (pp. 278–285). Springer, Berlin, Heidelberg.

[47] Hsu CW, Lin CJ. A comparison of methods for multi-class support vector machines. IEEE transactions on Neural Networks. 2002 8 7;13(2):415–25. 10.1109/72.991427 18244442

[48] Ben-Hur A, Weston J. A user’s guide to support vector machines. In Data mining techniques for the life sciences 2010 (pp. 223–239). Humana Press. 10.1007/978-1-60327-241-4_13 20221922

[49] Hsu CW, Chang CC, Lin CJ. A practical guide to support vector classification. Technical Report. Department of Computer Science and Information Engineering. University of National Taiwan. Taipei. 2003; pp. 1–12.

[50] Lior R. Data mining with decision trees: theory and applications. World scientific; 2014 9 3.

[51] Opitz D, Maclin R. Popular ensemble methods: An empirical study. Journal of artificial intelligence research. 1999 8 1; 11:169–98.

[52] Polikar R. Ensemble based systems in decision making. IEEE Circuits and systems magazine. 2006 9 6;6(3):21–45.

[53] Breiman L. Bagging predictors. Machine learning. 1996 8 1;24(2):123–40.

[54] Breiman L. Bias, variance, and arcing classifiers. Tech. Rep. 460, Statistics Department, University of California, Berkeley, CA, USA; 1996 4.

[55] Zhou ZH. Ensemble methods: foundations and algorithms. CRC press; 2012 6 6.

[56] Kearns M. Thoughts on hypothesis boosting. Unpublished manuscript. 1988 12; 45:105.

[57] Kearns M, Valiant L. Cryptographic limitations on learning Boolean formulae and finite automata. Journal of the ACM (JACM). 1994 1 2;41(1):67–95.

[58] Ho T.K., 1998. The random subspace method for constructing decision forests. IEEE transactions on pattern analysis and machine intelligence, 20(8), pp.832–844.

